# Analysis of semaglutide's effect on Alzheimer's disease risk

**DOI:** 10.1002/alz.14462

**Published:** 2024-12-31

**Authors:** Chi‐Kuei Hsu, Chih‐Cheng Lai

**Affiliations:** ^1^ Department of Internal Medicine E‐Da Hospital I‐Shou University Kaohsiung Taiwan; ^2^ School of Medicine for International Students College of Medicine I‐Shou University Kaohsiung Taiwan; ^3^ Department of Intensive Care Medicine Chi Mei Medical Center Tainan Taiwan

1

To the Editor,

A recent study^1^ investigating the effects of semaglutide on Alzheimer's disease (AD) risk in patients with type 2 diabetes (T2D) presents intriguing findings, yet several methodological considerations warrant careful examination. While the study contributes valuable insights to the growing body of evidence regarding semaglutide's potential neuroprotective effects, certain limitations merit further discussion to guide future research endeavors.

The study's primary methodological challenge lies in its treatment of semaglutide exposure. The analysis does not adequately account for crucial variables such as medication dosage and duration of use. In clinical practice, semaglutide dosing varies significantly among patients, often requiring individualized titration based on glycemic control and tolerability. Understanding dose–response relationships and temporal effects could provide valuable insights into the therapeutic potential of semaglutide. Moreover, the duration of exposure needed to achieve potential neuroprotective effects remains unclear. This limitation significantly impacts the interpretation of the observed benefits and their clinical applicability, as optimal dosing strategies for AD prevention may differ from those established for glycemic control.

The matching methodology, while comprehensive for many parameters, overlooked several significant modifiable risk factors for AD. Physical inactivity, excessive alcohol consumption, social isolation, low educational engagement, and limited cognitive stimulation are well‐established risk factors that could confound the relationship between semaglutide use and AD risk.^2^, ^3^, ^4^, ^5^ These lifestyle factors not only influence AD risk directly but may also affect medication adherence and overall health outcomes. Furthermore, although the prevalence of T2D complications was well balanced between groups, the status of T2D control, as measured by HbA1c levels, remained unknown. Given that poor glycemic control is associated with cognitive decline, this represents a significant unmeasured confounder that could affect the study's conclusions.

The complexity of AD pathogenesis, particularly in T2D patients, necessitates consideration of multiple comorbidities. AD risk increases with the number of underlying conditions,^6^ yet the study did not employ a standardized comorbidity index. Incorporating validated tools such as the Charlson Comorbidity Index or Elixhauser Comorbidity Index would strengthen the analysis by accounting for the cumulative effect of multiple conditions on AD risk. This approach would provide a more nuanced understanding of the relationship between semaglutide use and AD risk in the context of overall patient health status.

Despite these limitations, the study makes valuable contributions to understanding the potential protective effects of semaglutide against AD in T2D patients. However, to establish more robust evidence, subsequent studies should incorporate several key methodological improvements. First, comprehensive dosage and duration data should be collected and analyzed to establish optimal treatment protocols. Second, future research should account for lifestyle‐related AD risk factors through detailed patient questionnaires or validated assessment tools. Third, measures of diabetes control, including HbA1c trajectories, should be incorporated into the analysis. Finally, implementing a validated comorbidity index would provide a more complete picture of patients’ overall health status and its impact on AD risk.

Future research addressing these limitations will be crucial in determining the true effectiveness of semaglutide in preventing AD among T2D patients and informing clinical practice guidelines. As the prevalence of both T2D and AD continues to rise globally, establishing robust evidence for potential preventive strategies becomes increasingly important for public health.

## CONFLICT OF INTEREST STATEMENT

The authors declare no conflict of interest.

## References

[1] Wang W , Wang Q , Qi X , et al. Associations of semaglutide with first‐time diagnosis of Alzheimer's disease in patients with type 2 diabetes: target trial emulation using nationwide real‐world data in the US. Alzheimers Dement. 2024;20(12):8661‐8672. 39445596 10.1002/alz.14313 PMC11667504

[2] Livingston G , Huntley J , Sommerlad A , et al. Dementia prevention, intervention, and care: 2020 report of the Lancet Commission. Lancet. 2020;396(10248):413‐446. 32738937 10.1016/S0140-6736(20)30367-6 PMC7392084

[3] Kivimäki M , Singh‐Manoux A . Prevention of dementia by targeting risk factors. Lancet. 2018;391(10130):1574‐1575. 10.1016/S0140-6736(18)30578-6 29695343

[4] Schwarzinger M , Pollock BG , Hasan OSM , Dufouil C , Rehm J , QalyDays Study Group . Contribution of alcohol use disorders to the burden of dementia in France 2008‐13:a nationwide retrospective cohort study. Lancet Public Health. 2018;3(3):e124‐e132. 29475810 10.1016/S2468-2667(18)30022-7

[5] Kuiper JS , Zuidersma M , Oude Voshaar RC , et al. Social relationships and risk of dementia: a systematic review and meta‐analysis of longitudinal cohort studies. Ageing Res Rev. 2015;22:39‐57. 25956016 10.1016/j.arr.2015.04.006

[6] Vassilaki M , Aakre JA , Cha RH , et al. Multimorbidity and risk of mild cognitive impairment. J Am Geriatr Soc. 2015;63(9):1783‐17490. 26311270 10.1111/jgs.13612 PMC4607039

